# Strong connectivity in real directed networks

**DOI:** 10.1073/pnas.2215752120

**Published:** 2023-03-16

**Authors:** Niall Rodgers, Peter Tiňo, Samuel Johnson

**Affiliations:** ^a^School of Mathematics, University of Birmingham, Birmingham B15 2TT, United Kingdom; ^b^Topological Design Centre for Doctoral Training, University of Birmingham, Birmingham B15 2TT, United Kingdom; ^c^School of Computer Science, University of Birmingham, Birmingham B15 2TT, United Kingdom; ^d^The Alan Turing Institute, British Library, London NW1 2DB, United Kingdom

**Keywords:** directed networks, feedback, percolation theory, strong connectivity, trophic incoherence

## Abstract

In many real, directed networks, the strongly connected component of nodes which are mutually reachable is very small. This does not fit with current theory, based on random graphs, according to which strong connectivity depends on mean degree and degree–degree correlations. And it has important implications for other properties of real networks and the dynamical behavior of many complex systems. We find that strong connectivity depends crucially on the extent to which the network has an overall direction or hierarchical ordering—a property measured by trophic coherence. Using percolation theory, we find the critical point separating weakly and strongly connected regimes and confirm our results on many real-world networks, including ecological, neural, trade, and social networks. We show that the connectivity structure can be disrupted with minimal effort by a targeted attack on edges which run counter to the overall direction. This means that many dynamical processes on networks can depend significantly on a small fraction of edges.

## Significance

Many real-world systems are connected in a complex directed network, such as food webs, social, or neural networks. Spreading and synchronization processes often occur in such systems, and understanding the percolation transition (formation of a giant connected component) is key to controlling these dynamics. However, unlike in the undirected case, this had not been understood in directed networks with realistic nonrandom architectures. We provide a universal framework in which the percolation threshold for networks to be strongly connected (every node to be able to reach every other) can be analytically predicted on any real-world network and verify this on a diverse dataset. This explains why many real, dense networks are not strongly connected, in contrast to random-graph theory.

Understanding the connectivity structure of a directed network is crucial in many different contexts. Can every node be reached in a communications network or one-way street grid? How will a disease spread or will a dynamical system be stable and resilient to perturbation? Whether a network will be connected has been well-studied in the case of undirected networks through percolation theory; however, this is less well-understood in directed networks, and hence real-world systems, which are often directed (1, 2). We demonstrate through understanding the global directionality and hierarchical organization of directed networks through a method known as Trophic Analysis (3) that it is possible to construct a phase diagram which predicts if real networks are strongly connected using only the average degree and the incoherence parameter which measures the global directionality going beyond previous understanding based on directed random graphs (4). The notion of global directionality provided by trophic analysis enables us to talk meaningfully of “forward” and “backward” edges and gives us an insight into the directed network which is not possible without this. It is the backward edges that break the overall hierarchical structure. This hierarchical structure can be found in almost all real-world networks, not just networks with obvious hierarchy such as food webs where the hierarchy is number of steps from the nodes of zero in-degree such as plants. Global directionality, Trophic Incoherence, has been linked to network nonnormality (3) which has been shown to be ubiquitous in real directed systems (5, 6). We use the insight that the strong connectivity is driven by edges which break the hierarchical ordering to apply percolation theory (7) to these “backward” edges and an analytical estimate of the number of such edges derived from the global directionality to analyze the connectivity structure and predict the threshold for the emergence of a giant strongly connected component. This provides an insight beyond degree (4) and explains why, even if they have high mean degree, highly structured networks like food-webs often have very small strongly connected components. We extend our understanding of strong connectivity to real networks beyond previous results on directed random graphs (4) which do not capture the complex structures of real-world systems. We demonstrate the role the “backward” edges have in controlling the strong connectivity by conducting a targeted attack on these edges. This removes the strongly connected component while maintaining the weak connectivity. We show the vital role that strong connectivity plays in dynamics, *SI Appendix*, by comparing the spread of an infection using SIS dynamics; synchronization of coupled Kuramoto Oscillators and how a new state establishes itself in the Majority Vote and Voter Models before and after the targeted attack. These dynamics demonstrate the role of hierarchy in the dynamics as the global directionality and “backward” edges drive feedback and how their removal creates an asymmetry in the ability of nodes to interact with each other dependent on their position in the hierarchy.

Trophic Analysis is a technique which is used to calculate the global directionality and the hierarchy in directed complex networks (3). Complex networks are graphs which represent real-world systems. Graphs are sets of vertices (nodes) and edges (links) which represent connections between elements in the system. Graphs are topological objects as they do not need to have a distance scale, they merely represent whether elements are connected. A directed graph (or digraph) is one in which the connections between elements go in only one direction. This is very common in real-world systems (2) which can be intrinsically directional like a prey–predator food web interaction or following a profile on social media. In complex networks, it is common to represent a graph via an adjacency matrix. For a graph consisting of *N* nodes, the adjacency matrix, *A*, is defined such that

[1] $$\begin{array}{c} A_{\mathrm{ij}}=\left\{\begin{array}{c} 1\;\;\text{if there exists an edge}\;i\to j\\ 0\;\;\text{otherwise} \end{array}\right.\;\;\;\;\;\;\;. \end{array}$$

As a result, the topology of the graph can be represented by the nonzero entries of this matrix. This form is preferred for studying complex networks as it is convenient for computer simulations, defining dynamical systems on the network, and network properties can easily be accessed from the properties of this matrix. In a directed graph, this matrix is not necessarily symmetric, *A*_*ij*_ ≠ *A*_*ji*_, since the interactions are only in one direction. Undirected graphs always have symmetric adjacency matrices. Adjacency matrices can also be weighted to capture the strength of an interaction. However, for simplicity, we focus here on the unweighted case. An undirected graph is connected if and only if for any pair of distinct nodes there exists a path connecting them. In directed graphs, the notion of connectivity is more complex. A directed graph is weakly connected if there is a path between all pairs of vertices when edge direction is ignored. A digraph is strongly connected if for every pair of nodes *i* and *j*, there is a directed path from *i* to *j* and another from *j* to *i* (in other words, every node is reachable from every other node).

It is common for real-world directed networks to be weakly connected, but many are not strongly connected. In such cases, the extent to which a network approaches strong connectivity can be quantified by the size of its largest strongly connected component (i.e., the largest subgraph which is strongly connected).

Later on, when we talk about predicting strong connectivity in real networks from a classification problem perspective, we use the more general definition of *α*-strong connectivity, which requires the largest strongly connected component to be larger than *α* times the number of nodes (0 <  *α* <  1). In our analysis, we set *α* = 0.9.

In an undirected graph, each node has a degree, corresponding to the number of edges connected to it. In a directed graph, the in-degree of a node *i* is the number of edges which point to it $k_{i}^{\mathrm{in}}=\sum_{j}A_{\mathrm{ji}}$ and its out-degree is the number of edges pointing to other nodes, $k_{i}^{\mathrm{out}}=\sum_{j}A_{\mathrm{ij}}$. If the adjacency matrix is transposed the in- and out-degrees swap.^*^

## Trophic Analysis.

Trophic Analysis first arose in the study of food webs (8). The hierarchical organization of the network, as measured by a property called trophic coherence, was proposed as a solution to May’s paradox regarding the stability of food webs (9). The name arises from the trophic level of a species in ecology (10). This definition relies on the existence of basal nodes, that is, nodes with in-degree zero. A new definition was then proposed in ref. 3 which removed this constraint and made it applicable to any directed network. We follow the new definition (3), although most previous work used the original convention (8).

Trophic Analysis has been used to study many aspects of directed networks, including the structure of food webs (11), spreading processes such as epidemics or signals in neural networks (12), resilience of infrastructure networks (13, 14), control of organizations (15), and networks in economics and finance (3).

Trophic Analysis is composed of two parts: the node level information, Trophic Level, and the global information, Trophic Incoherence (3). Trophic level gives a measure of where a node sits in the hierarchy of a directed network. For example, in a food web, plants would be the low trophic level nodes and carnivores the high trophic level nodes, as energy flows up the food web from low to high trophic level. This can however be generalized to any directed network. Trophic levels are calculated by solving the *N* × *N* matrix equation given by

[2] $$\begin{array}{c} \Lambda h=v, \end{array}$$

where *h* is the vector of trophic levels and the “imbalance” vector is the difference between in- and out-degrees: *v* : *i* = *k*_*i*_^*in*^ − *k*_*i*_^*out*^. *Λ* is the Laplacian matrix,

[3] $$\begin{array}{c} \Lambda =\;\text{diag}\left(u\right)-A-A^{T}, \end{array}$$

where *u* is the sum of in- and out-degrees, *u*_*i*_ = *k*_*i*_^*in*^ + *k*_*i*_^*out*^, *A* is the adjacency matrix and *A*^*T*^ its transpose. These are all quantities which can be simply evaluated from the adjacency matrix. Note that Eq. **2** cannot be solved by inverting *Λ* since this matrix is singular. However, one can use other methods, such as LU decomposition, and for large networks, the equation can be solved iteratively.

Moreover, Eq. **2** is invariant under the addition of a constant vector to *h*. We therefore follow the convention that the lowest level node takes the value *h* = 0 (3).

Trophic Incoherence measures the global directionality of the network (3), based on the distribution of level differences across edges. If the network is maximally coherent, the edges only connect to nodes exactly one level above them and the network is perfectly hierarchical and globally directed. If the network is highly incoherent then the edges connect without respect to the levels and there is no global directionality. This is quantified via the trophic incoherence parameter *F* which is defined as

[4] $$\begin{array}{c} F=\frac{\sum_{\mathrm{ij}}A_{\mathrm{ij}}{\left(h_{j}-h_{i}-1\right)}^{2}}{\sum_{\mathrm{ij}}A_{\mathrm{ij}}}. \end{array}$$

This equation measures, averaged over the system, the square of the deviation in level difference of destination to source vertex from 1 across the edges of the graph. This equation is bound between 0 and 1 (3). Networks with *F* = 0 are perfectly coherent, they have distinct integer levels in which all nodes are placed, and they are acyclic. When *F* = 1, every node has the same level and the network has no hierarchy. Examples of *F* = 1 networks are directed cycles. Networks which have *F* = 1 are perfectly balanced (*k*_*i*_^*in*^ = *k*_*i*_^*out*^ for all nodes *i*) and are therefore unlikely to come about from a fully random process. For example, random graph models such as the Erdős-Rényi model (16, 17) lead to networks where *F* is around 0.95, depending on sparsity.

The trophic levels can be thought of as the set of values, *h*, which minimize *F* (3) for a given *A*. This leads to Eq. **2**. It is also possible, equivalently, to speak in terms of Trophic Coherence, which can be defined as 1 − *F*. When we say “top of the hierarchy,” we mean the nodes with high trophic level, and when we use the phrase “bottom of the hierarchy,” we mean nodes of low trophic level.

The definition of *h* as a measure of node hierarchy has been proposed independently more than once. For example, SpringRank (18) uses a physical argument to arrive at the same minimization function as Trophic Analysis but without the same quantification of the global directionality (3). It is also possible to use a Helmholtz–Hodge decomposition to construct the idea of levels and “circularity” (19, 20), which leads to a different set of terms to quantify hierarchy and directedness, and it can be shown that this method is equivalent to Trophic Analysis (3).

## Generated networks.

When we require numerically generated networks to better sample the full range of Trophic Incoherence and degrees, we use the same variant of the generalized preferential preying model as ref. 21, which was based on work from ref. 12. This model allows the Trophic Incoherence of a generated network to be approximately controlled. This is done by a taking an initial network structure and adding edges with a probability which is proportional to the level difference between the nodes, in a way which is determined by a “temperature” parameter. This probability is defined as

[5] $$\begin{array}{c} P_{\mathrm{ij}}\propto \exp\left[-\frac{{\left({\tilde{h}}_{j}-{\tilde{h}}_{i}-1\right)}^{2}}{2T_{\;\text{Gen}}^{2}}\right], \end{array}$$

where ${\tilde{h}}_{i}$ is the temporary trophic level assigned during the generation. *T*_*Gen*_ is the generation temperature used to control the incoherence. At high *T*_*Gen*_, edges are added without respect to the level structure so it produces an incoherent network similar to a random graph; while at low temperature, the edges are only added when the level difference is near one, producing a very coherent network. Details of how to efficiently sample the possible edges and generate networks in this way can be found in ref. 21.

## Results

### Fraction of Edges Going Against the Hierarchy.

It is possible to analytically estimate the number of edges that go “backward”—i.e., against the hierarchy. We define a backward edge as one where the difference between the trophic level of the target vertex minus that of the source vertex is nonpositive. As we go on to see, these edges are important as they determine the strong connectivity of the network, because they are needed to induce a path back down the hierarchy. This fraction of edges is useful in further calculation of strong connectivity.

The first part of this derivation is to assume that the edges follow an approximately Gaussian distribution in trophic level differences (17), where the mean is the mean level difference, $\bar{z}$, and the standard deviation is given by $\bar{z}\eta$ (3). We assume that the level differences follow a Gaussian distribution as this assumption was used to derive the results linking trophic coherence to spectral radius (17), which also hold for the new definition of trophic levels and coherence (3, 21). In addition, we have observed that for many real networks (some examples are given in *SI Appendix*), the distribution of level differences can be well-approximated by a Gaussian. Other formulations of hierarchy make similar assumptions, for example, when SpringRank was first introduced (18) it was assumed that the ranks followed a Gaussian distribution and shown that adding a quadratic regularization term is equivalent to a Gaussian prior on the ranks.

The mean level difference can be computed from the Trophic Incoherence,

[6] $$\begin{array}{c} \bar{z}=1-F, \end{array}$$

which was derived in ref. 3 by writing the function for Trophic Incoherence as a function of the mean and SD of the level differences and then minimizing it. The parameter *η* which is the SD scaled by the mean trophic level difference can also be expressed in terms of *F* (3) by similarly writing *F* as a function of the SD and mean level differences

[7] $$\begin{array}{c} \eta =\sqrt{\frac{F}{1-F}}. \end{array}$$

Note that Eqs. **6** and **7** hold for any digraph, and are not dependent on the assumption of Gaussian differences.

Assuming that the edge level differences, *x*_*ij*_ = *h*_*j*_ − *h*_*i*_, follow a Gaussian distribution leads to the probability distribution,

[8] $$\begin{array}{c} p\left(x_{\mathrm{ij}}\right)=\frac{1}{\bar{z}\eta \sqrt{2\pi }}\exp\left[-\frac{1}{2}{\left(\frac{x_{\mathrm{ij}}-\bar{z}}{\bar{z}\eta }\right)}^{2}\right]. \end{array}$$

The fraction of edges which do not go in the same direction as the hierarchy is the integral of this distribution from negative infinity to 0. The cumulative distribution of a Gaussian is well-known and the result can be written in terms of the error function as

[9] $$\begin{array}{c} \bar{\beta (F)}=\frac{1}{2}\left[1+\;\mathrm{erf}\;\left(-\frac{1}{\sqrt{2}}\sqrt{\frac{1-F}{F}}\right)\right], \end{array}$$

where we have substituted for $\bar{z}$ and *η* in terms of *F*. Hence, $\bar{\beta (F)}$ can be regarded as the expected fraction of backward edges under the assumption of Gaussian-distributed trophic differences. This equation can be understood by looking at the limiting cases where *F* equals 1 or 0. When F approaches 1, the error function goes to zero and then half the edges go against the “hierarchy,” as every node approaches the same level and hence there is an equal likelihood of going forward or backward. When *F* = 0, the error function goes to negative 1 so the expression cancels and no edges go backward, which makes sense as the network is fully coherent. Due to the fact that edges of level difference zero are counted as backward, the approximation breaks down in the extreme case of a perfectly balanced network such as a directed cycle or undirected graph, as all the edge differences are zero. Hence, the measurement labels all the edges as backward, whereas the approximation limits to half the edges going backward.

**Fig. 1.**
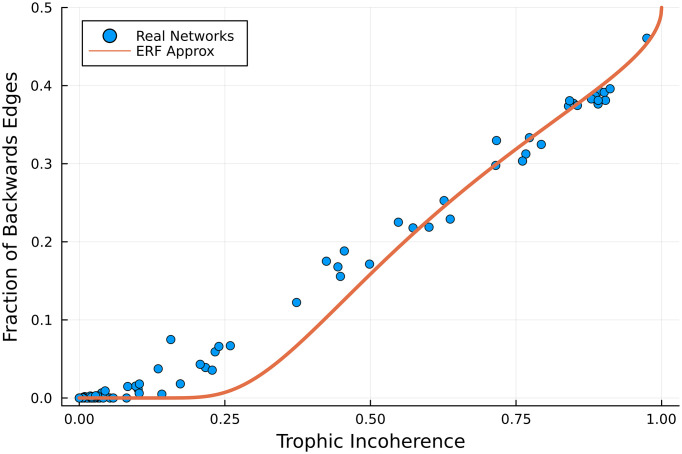
Number of backward edges in various real networks (symbols) and the prediction of Eq. **9** (line), against trophic incoherence *F*. Data from ref. 25 and 22 (original sources in *SI Appendix*).

This prediction holds well in real networks, as shown in Fig. 1, with some small deviations. This is likely because of the assumption that the distribution of edge differences is Gaussian. The relationship between *F* and the number of backward edges looks almost linear, but the nonlinearity at low *F* is important: It is possible for a network without backward edges not to be maximally coherent, since certain feed-forward motifs generate some incoherence (3).

All the real networks used in this paper and the original sources can be found in *SI Appendix*. For convenience, we cite the online sources in the main text. This includes all the networks used in ref. 17, plus a sample of networks from ref. 22. This dataset includes metabolic networks, neural networks, trade networks, food webs, and social networks. The number of backward edges could also provide a rough estimate of the upper bound on the size of a feedback arc set, the number of edges which need to be removed to make the graph acyclic (23). The link between hierarchy-breaking edges and a heuristic to approximate the cycles has been made before (23) with different measures of the hierarchical ordering, including PageRank (24); however, these lack an analytical estimate of the expected number of backward edges. The probability of a path going backward was also used to derive various expressions in the “coherence ensemble” of random graphs (17). However, none of these works established the link to strong connectivity and the emergence of a giant strongly connected component, which follows.

### Derivation of Strong Connectivity Critical Point and Phase Diagram.

It is possible to derive an estimate of the percolation transition threshold for the emergence of a giant strongly connected component in directed networks using the insight gained from the hierarchical structure. This can be done by observing that if the nodes in a network are ordered in some way, then the edges which break that ordering by going “backward” are the important edges for strong connectivity. Adding more edges in the forward direction will not make the network strongly connected if it is very strictly hierarchical, as they do not provide a way to move back down the ordering toward the bottom of the network. In this way, the growth of the strongly connected component in a directed network can be thought of as a percolation process on the backward edges, where the backward edges connect the layers of the network. This makes it possible to move back down the hierarchy, thereby creating a giant strongly connected component.

This can be expressed using the framework for solving percolation problems set out in ref. 7. This framework decomposes the percolation process to transitions between l-step neighborhoods, which in our case can be thought of as steps down the hierarchy. We assume a weakly connected network when the backward edges are removed, which is a reasonable assumption for real networks as the fraction of backward edges is usually small and the networks are dense enough so that removal of the backward edges does not result in the network being disconnected. It is possible to find counter examples to this where the network does become disconnected if the backward edges are removed (Section A). This, however, only occurs when the backward edges are calculated once and trophic level is not recalculated after each removal. This is proven in Section A, where we also show examples of the maintenance of weak connectivity in real networks, justifying this assumption. We wish to find the percolation threshold for the network to be at least weakly connected by only backward edges and hence have a giant strongly connected component.

We define a directed subgraph, *G*(*V*, *E*_*B*_), made up only of backward edges, where the trophic level difference is less than or equal to 0, of the larger graph *H*(*V*, *E*) containing all the edges from which the trophic levels are calculated. Following the steps laid in out in ref. 7, we introduce the *l*-neighborhood of a vertex, *y*. This is recursively defined as

[10] $$\begin{array}{c} N_{l}(y)=\underset{X\in V(N_{l-1}(X))}{\cup }N_{1}(X), \end{array}$$

where *V*(𝒩_*l* − 1_(*X*)) is the set of all of the vertices within the neighborhood 𝒩_*l* − 1_(*X*). This neighborhood can be thought of as the nodes reachable in within *l* steps from vertex *y*, illustrated in more detail in ref. 7. The percolation transition can then be understood by analyzing the surfaces of these neighborhoods, which can be defined as the vertex sets

[11] $$\begin{array}{c} V_{l}:=V\left(N_{l}\left(y\right)\right)\setminus V\left(N_{l-1}\left(y\right)\right). \end{array}$$

These are the nodes which lie exactly *l* steps from the origin vertex, *y*. This origin vertex can in general be any vertex, but in the case of backward connectivity, we choose the vertex with the highest trophic level. Following the work of ref. 7, the system is above the percolation threshold if

[12] $$\begin{array}{c} \underset{l\to \infty }{\lim}E[o(V_{l})]>0, \end{array}$$

where 𝔼[*x*] is the expectation value of *x* and *o*(*V*_*l*_) is the number of connected nodes on the surface *l*. The expectation values are taken using draws from the “coherence ensemble,” the set of all unweighted directed networks of fixed trophic coherence, size, and degree distribution, used in refs. 2, 17 and 3. Eq. **12** can be understood to mean that there is a giant connected component if the expectation value of a node being connected is greater than zero as the surface size extends to infinity. This is analogous to the probability of a branching process not dying out as the number of steps tends to infinity.

Our assumption for our specific case is that in the network of backward edges, the expected number of connected nodes in a surface is simply the number of connected nodes in the previous surface multiplied by the average number of backward connections. This is

[13] $$\begin{array}{c} E\left[o\left(V_{l+1}\right)\right]=\beta \left\langle k\right\rangle E\left[o\left(V_{l}\right)\right], \end{array}$$

where ⟨*k*⟩ is the mean total degree and *β* is the fraction of edges which go backward. This equation can then be solved iteratively assuming that 𝔼[*o*(*V*_0_)] = *C*, where *C* is some finite constant representing the number of nodes at the top of the hierarchy. This leads to

[14] $$\begin{array}{c} E\left[o\left(V_{l}\right)\right]={\left(\beta \left\langle k\right\rangle \right)}^{l}C. \end{array}$$

Taking the limit *l* goes to infinity leads to the result

[15] $$\begin{array}{c} \lim_{l\to \infty }E\left[o\left(V_{l}\right)\right]=\left\{\begin{array}{c} \infty \;\;\text{if}\;\beta \langle k\rangle >1\\ C\;\;\text{if}\;\beta \langle k\rangle =1\\ 0\;\;\text{if}\;\beta \langle k\rangle <1 \end{array}\right.. \end{array}$$

This means that we expect a giant strongly connected component when *β*⟨*k*⟩> 1, which means that on average each node has at least one backward connection. This can also be written directly as a function of trophic incoherence using the expected value of *β*,

[16] $$\begin{array}{c} \frac{\left\langle k\right\rangle }{2}\left[1+\;\mathrm{erf}\;\left(-\frac{1}{\sqrt{2}}\sqrt{\frac{1-F}{F}}\right)\right]>1. \end{array}$$

**Fig. 2.**
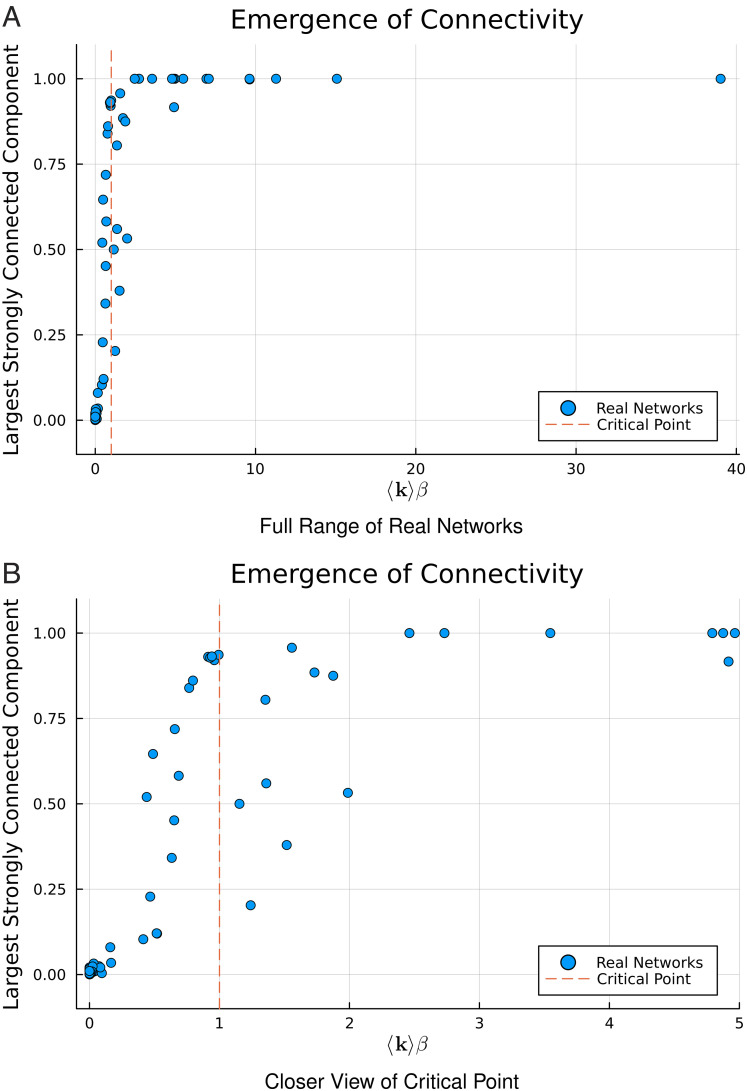
Fraction of nodes in the largest strongly connected component against *β*⟨*k*⟩ for several real networks. The critical point, *β*⟨*k*⟩=1, is indicated via a vertical line, Panel *A*. Panel *B* shows only the networks with *β*⟨*k*⟩≤5. Data from refs. 25 and 22 (original sources in *SI Appendix*).

This estimate, which uses very little information about the network structure (only the mean degree and trophic incoherence), works well for real networks, Fig. 2 *A* and *B*. This result shows how the understanding of hierarchy can allow insights into the connectivity of directed networks. The result relies upon the ability to calculate the hierarchy for any directed network, and the realization that the backward nodes shape connectivity and that their number can be linked to the global directionality and analytically estimated. Other measures of hierarchy would allow the number of “backward” edges to be enumerated numerically but lack the link to global directionality which gives the intuition behind these results.

The equation for the percolation threshold, under the Gaussian edge difference assumption, in terms of *F*, Eq. **16**, can be expressed as an equation for the critical incoherence. This can be written as

[17] $$\begin{array}{c} F_{c}={\left[1+2{\left({\;\mathrm{erf}\;}^{-1}\left(\frac{2}{\left\langle k\right\rangle }-1\right)\right)}^{2}\right]}^{-1}. \end{array}$$

**Fig. 3.**
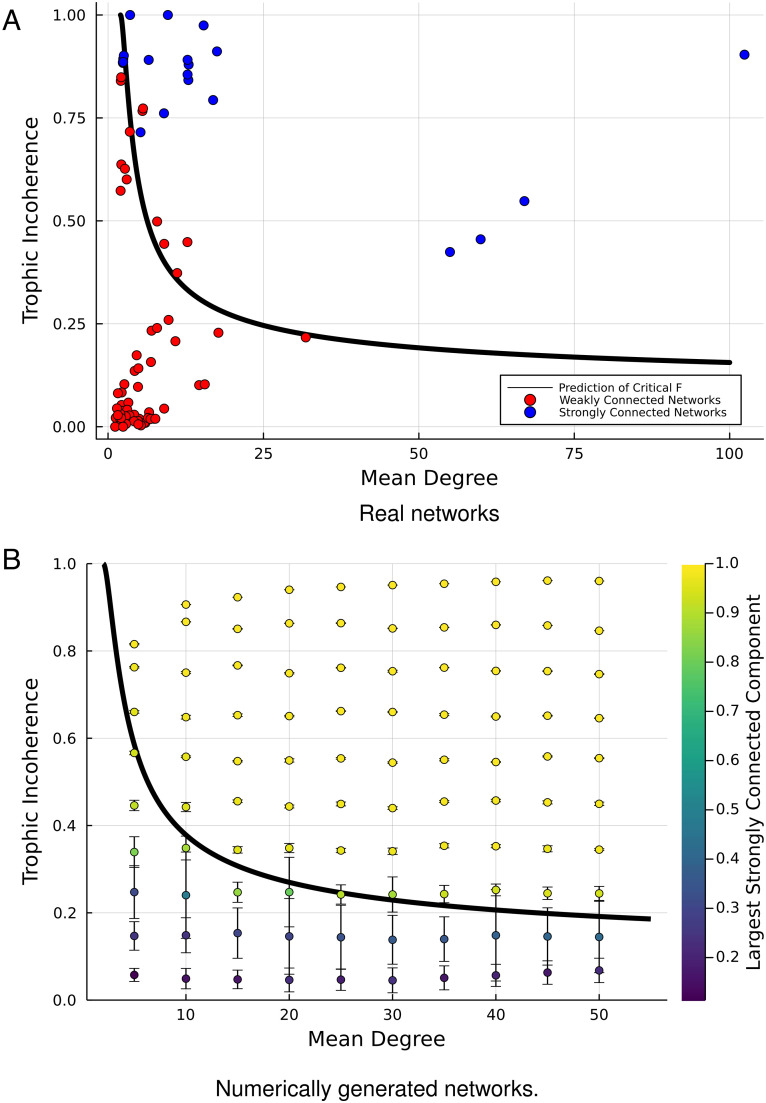
Prediction of strong connectivity using the trophic incoherence (y-axis) and mean degree (x-axis), based on the critical incoherence *F*_*c*_ given by Eq. **17**. Panel (*A*): Real networks from refs. 25 and 22 (original sources in *SI Appendix*). Panel (*B*): 1,000 Networks with *N* = 500 generated numerically as in ref. 21, with varying mean degrees and binned by trophic incoherence. Error bars are one SD.

For a fixed ⟨*k*⟩, if *F* is greater than the predicted value, then we expect the network to be strongly connected. This allows the existence of a giant strongly connected component of a real network to be predicted based only on *F* and the average degree. The accuracy of this prediction for real networks is demonstrated in Fig. 3*A*, where the prediction of strong connectivity is formulated as a classification problem. We assume that the positives in our sample are the *α*-strongly connected networks which have a strongly connected component of at least 90% of the network size, and the negatives are the networks which fall below this value. The confusion matrix for this process reads as True Positive Rate 0.783, True Negative Rate 0.906, False Negative Rate 0.217, False Positive Rate 0.09375. This is quite a good classification rate as we note and demonstrate in Fig. 3*A* that all the errors lie close to the transition line where we do not expect to be able to classify the networks with a high level of accuracy into the two categories. We note, however, that the classification problem approach is very sensitive to the difficulty of the data chosen. For example, if a dataset were selected with very few networks in the intermediate region (say, a single network type, such as food webs), then the results would improve without any change in the method. In our dataset, we have 64 networks with a strongly connected component below 90% and 23 with a strongly connected component larger than this.

The only regions where the prediction is less good are close to the boundary; however, this is not surprising as we are not taking account of any finite size effects or potentially heterogeneous degree distributions and in particular how the backward edges are distributed. These results are broken down by network type in *SI Appendix*.

We can give further insight into the accuracy of the prediction by using numerically generated networks to better sample the parameter space and verify the results in a larger region. This is shown in Fig. 3*B*. We take 1,000 networks where *N* = 500, generated as in ref. 21, where each node has at least in-degree 1 which would make the trophic level impossible to calculate in the original definition from ecology (8), and then bin them by Trophic Incoherence and average the size of the strongly connected component. This result agrees well with the analytical prediction, with the networks well above the boundary being strongly connected and a large component forming in the networks around the boundary as expected.

These results, which hold for both real and generated networks and are based on the assumption of Gaussian edge differences, give a good insight into how global directionality determines strong connectivity and the emergence of a giant strongly connected component. Even for a very large mean degree, a network is still unlikely to be strongly connected if *F* is low enough, which demonstrates that more than just information on node degrees is needed for estimating the connectivity of a directed network. Why some real networks lie close to the transition line and properties of networks at this point may be a possible avenue for future work.

The value of this analysis can be highlighted by comparing to the results obtained by taking this real network dataset and trying to predict the emergence of a giant strongly connected component without using the hierarchical structure. This can be roughly estimated using results from refs. 4 and 17, where one would expect the strongly connected component to grow very quickly and the percolation to occur when the branching factor is greater than 1,

[18] $$\begin{array}{c} \frac{\left\langle k^{\;\text{in}}k^{\;\text{out}}\right\rangle }{\left\langle k\right\rangle }>1. \end{array}$$

**Fig. 4.**
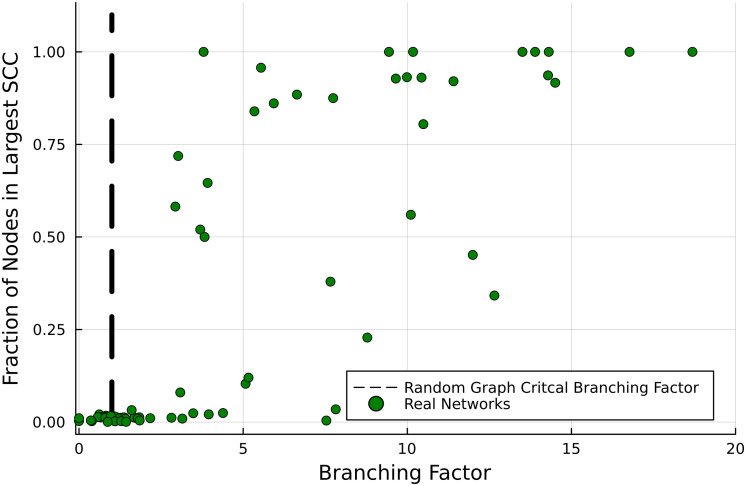
Prediction of strong connectivity based on the branching factor for real networks. Contrast with Fig. 2*A*, based on trophic coherence as presented here. Data from refs. 25 and 22 (original sources in *SI Appendix*).

This is demonstrated in Fig. 4, which shows how many networks of very high branching factor nevertheless have a small strongly connected component. The figure represents a closer look at the critical point, and networks of very high branching factor can be found in the supplementary information. This demonstrates how the directional organization is a vital part of the connectivity structure of real networks. Understanding the interplay between global directionality (trophic incoherence) and ordering (trophic level) provides an intuition greater than each individual notion can.

For comparison, we also repeat the same classification experiment using the branching factor to predict if a network has a large strongly connected component. The confusion matrix for this process is True Positive Rate 1.0, True Negative Rate 0.219, False Negative Rate 0.0, False Positive Rate, 0.781. This is expected as such a classification technique predicts that almost every network apart from those of very small branching factor is strongly connected, which explains the very high true positive rate, but also the very high rate of false positives. This further quantifies why in order to understand how strong connectivity arises in real directed networks, it is important to factor in the global directionality of the system.

### Targeted Attacks on Backward Edges.

**Fig. 5.**
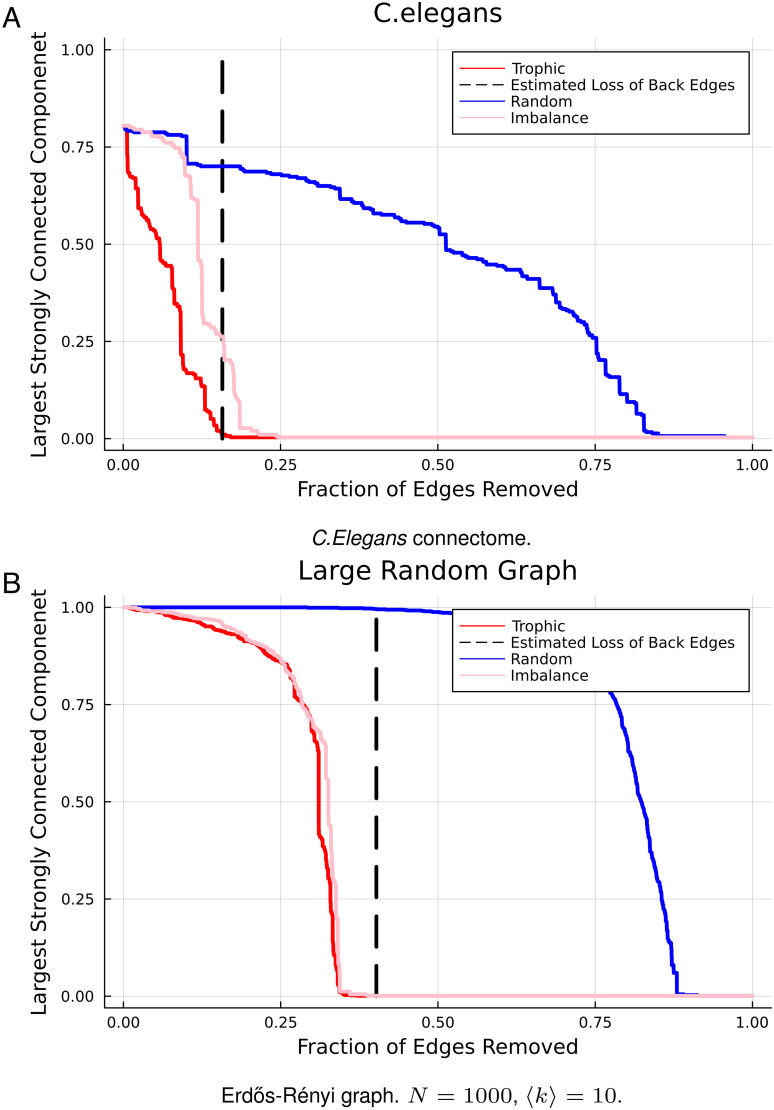
Size of the strongly connect component as edges are removed randomly, in order of trophic level difference, and in order of degree imbalance difference, for real and Erdős-Rényi networks (panels *A* and *B*, respectively). The dashed vertical lines represent the point beyond which no backward edges are predicted by Eq. **9**.

To demonstrate how the strong connectivity of a network depends on the edges which break the hierarchy, we can conduct a targeted attack on those edges and compare the degradation of the strongly connected component to that observed in a random attack. We remove edges in order of their trophic difference, starting by removing the edges with the most negative trophic difference. This only takes into account the hierarchical organization; it may be possible to destroy the strongly connected component faster using a different method, for example, attacking bottleneck edges or specifically trying to target edges breaking the hierarchy in different components of the network. However, when all the backward edges are removed, all cycles are destroyed and the strongly connected component is guaranteed to vanish. This can be demonstrated in real networks, Fig. 5*A*, such as the connectome of the worm *C. Elegans*. The point at which we estimate the “backward” edges to vanish, shown by the dashed line in Fig. 5*A*, is analytically estimated from Eq. **9** and predicts well the point at which the strongly connected component vanishes completely. We compare the attack using backward edges with two alternative attack strategies: 1) completely random edge attack, and 2) attack based on the edge degree imbalance differences, $\left(k_{j}^{\mathrm{in}}-k_{j}^{\mathrm{out}}\right)-\left(k_{i}^{\mathrm{in}}-k_{i}^{\mathrm{out}}\right)$. Here, we attack the most negative of degree imbalance differences as a proxy for trophic level. The intuition is that we roughly expect nodes of high in-degree and low out-degree to be high-level nodes and the inverse to be low-level nodes. We observed in the structured network of *C. Elegans* that the backward edges attack strategy is significantly better than the random attack. The imbalance strategy is better than random but is less successful than the trophic level strategy as it does not encompass the range of structural information captured in the trophic levels.

Similar results can be found for networks where there is no expectation of this kind of organized structure, like a dense random graph. This is shown in Fig. 5*B*. The figure shows that even in networks where there is little structure expected, there still exists a degree of directional organization that can be exploited to break down the network, as demonstrated by the comparison of the backward attack to the random attack. The number of edges needed to make the network acyclic and have no strongly connected component depends on the total number of backward edges, which is a function of the trophic incoherence and the mean degree, as in the percolation transition above. This demonstrates that the strongly connected component can be understood by focusing on the backward edges, which was the intuition that motivated the above derivation.

In the case of a random graph, because it lacks an overall hierarchical structure, the degree imbalances act as a good proxy for trophic level. Hence, an attack on the imbalances performs similarly to trophic level. A similar effect has been shown in ref. 26, where the success of different measures such as imbalance and PageRank was compared to trophic level, as incoherence varied, in predicting strategy choice in generalized rock–paper–scissors dynamics.

One important thing to note about this targeted attack is that it does not generally affect the size of the weekly connected component until all the backward edges are removed and then it behaves in the same way as a random attack in most real-world cases. For more details, Section A. This could be useful in situations where one wishes to attack the strongly connected component without disrupting the weakly connected component, which would happen if bottleneck edges were attacked, for example. This means that the backward edges can act as an approximation for the feedback arc set (23). The trophic level may not perform as well as specialist methods at this task (23) but does provide an analytical estimate of how many edges one would expect to need to remove in any large network, which can be simply calculated from *F*. It also explains why certain networks are more difficult to render acyclic based on where they lie on the phase diagram.

## Discussion and Potential Applications

There are a wide range of network applications where percolation in networks has been observed to be important (1). We highlight a few areas where our work may be useful, but this is not necessarily exhaustive. Strong connectivity and percolation can play an important role in city planning as networks of one-way streets must be strongly connected (27).

Trophic analysis can be useful for understanding spreading processes where the network is directed and there is some ordering to the network structure, for instance in ecological settings (28). A real-world example of this is the spreading of crown-of-thorns starfish on coral reefs (29–31). These starfish are a pest which eat coral reefs and can damage ecosystems. Outbreaks are governed by the spread of their larvae by the ocean currents. This process is directed as the larvae move in the direction the ocean flows. This is the kind of process Trophic Analysis could lend itself to as it could be used to understand the global connectivity structure to see if the outbreak is likely to spread across the reef or in a directed manner. It can be used to extend the existing analysis of a region’s vulnerability to outbreaks or danger as a starting point beyond simply the size of the out- and in-components (29), by factoring in where reefs sit in the network hierarchy determined by trophic level.

Our work could also relate to the growth of biological neural networks and formation of a giant strongly connected component of cells (32, 33). These neurons have previously been grown in circumstances where there is limited hierarchical structure and the degree distributions are well known. However, if the cells were exposed to a directed gradient, or in a real-life system are more likely to grow in a particular direction, Trophic Analysis may play a role in explaining this percolation threshold.

Our results may also partially explain the difference in percolation thresholds for dynamical processes on directed networks (34) compared to the undirected case, due to the effect of hierarchical ordering increasing the threshold for strong connectivity. This will be observed even in random graphs, as trophic incoherence does not usually reach one. The percolation of the strongly connected component and the direction of flow and spread of information may also play a role in communication networks and control and decision-making in organizations (15).

In general, Trophic Analysis can be used to modify the dynamics by understanding the hierarchical organization and the effect of localized perturbations, as well as highlighting the role of hierarchy-breaking edges in driving strong connectivity, feedback, and resilience in complex systems. Trophic Analysis is also a useful method due to the simplicity of the calculation and its interpretability, as the notion of directionality and place in hierarchy is quite simple and intuitive. This makes the method attractive to be employed in many settings as the barrier to entry is relatively low, while still providing good intuition into the structure of a directed network. Additionally, it provides motivation to focus on the intrinsic directional aspects of real-world systems, which are understudied compared to the undirected case (2).

Our results for strong connectivity, though quite robust, are based on a very simple approximation of the percolation of backward edges. So it may be possible to extend or repeat this result with a different measure of hierarchy or more information about the degree distribution, providing greater accuracy.

## Conclusion

We have shown how, by using Trophic Analysis to study the hierarchical ordering and global directionality of a directed network, it is possible to analytically estimate the number of “backward” edges which break this ordering and predict the threshold for the network to be strongly connected. From this, a phase diagram of strong connectivity in terms of trophic incoherence and mean degree can be derived which holds well for real directed networks. This shows that strong connectivity in directed networks is driven by more than just the degrees, and that hierarchy can play a significant role.

We highlight these results by conducting a targeted attack on “backward” edges, revealing their crucial role in maintaining a strongly connected component. In *SI Appendix*, we further illustrate the importance of these edges by implementing several dynamics (voter model, SIS, and Kuramoto oscillators) where the behavior is dictated by the strongly connected component and the trophic level of the initiating node.

## A. Removal of Backward Edges and Weak Connectivity

**Fig. 6.**
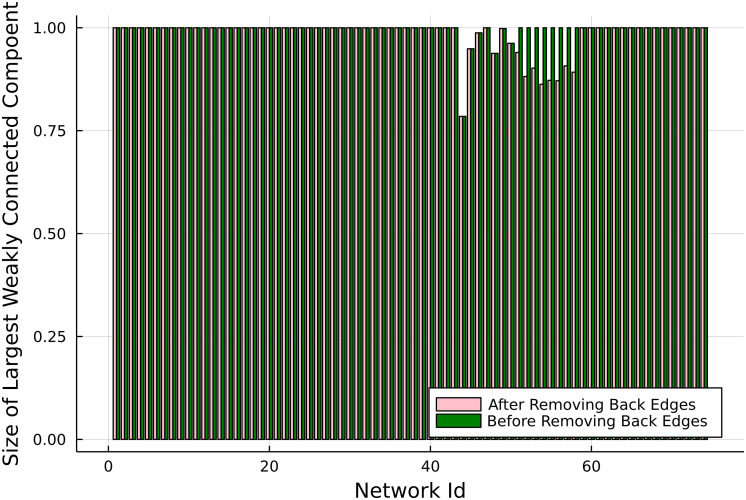
Lack of change in the size of the weakly connected component as the backward edges are removed from real networks (25) (original sources in *SI Appendix*).

Backward edges are in general linked to cycles and reciprocal edges; this means that in general, removing one backward edge is unlikely to separate a large, real network into distinct components. This explains why the size of the weakly connected component is generally unaffected when the backward edges are removed in real networks, Fig. 6.

**Fig. 7.**
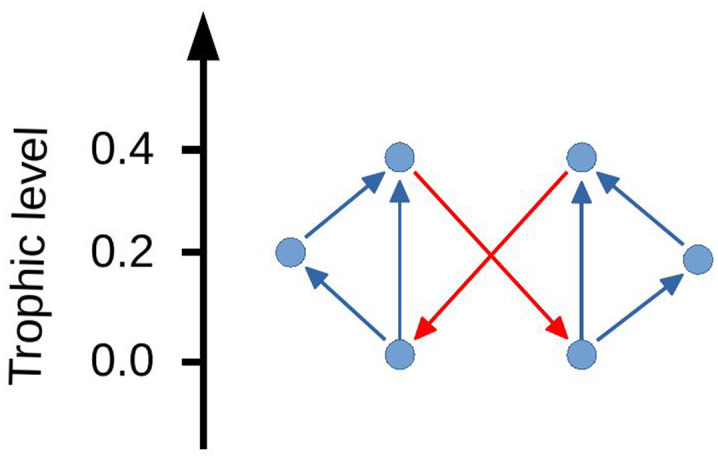
Example of network which is connected by two edges which go backward in Trophic level.

However, there are specific structures composed of interlocked cycles which can cause the network to become disconnected if the trophic level is calculated only once and then all the edges which are initially backward are removed as shown by the example in Fig. 7 where the backward edges are highlighted in red. This however is a specific case and does not seem to be found when studying the connectivity of real networks. In addition, if the trophic levels were recalculated after the first edge was removed, the second edge would then become forward. This leads to the property that if the trophic level is recalculated, then the network will not become disconnected. This is proved below.

Given an initial graph G(V,E), at each step remove the edge which is most backward in the trophic level and then recalculate the trophic level. Stop when all the edge differences are nonnegative. In order to break the network into separate components via this method, there would require a situation where the graph has been separated into two disjoint components joined by a single edge which upon removal would break the graph into disconnected pieces. If the trophic levels are recalculated, then it is never optimal for this edge to go backward in the trophic level as the levels of one of the components can be modified by a constant which will not change the global coherence apart from across the joining edge where it will become positive. If the edge is backward, it is always possible to reduce incoherence in this way so the configuration cannot exist upon recalculation.

## Supplementary Material

Appendix 01 (PDF)

## Data Availability

All study data are included in the article and/or *SI Appendix*. Previously published data were used for this work (25). All graph manipulations were carried out using Julia Package Graphs.jl (35). Code and data used as part of this study can also be found on Github (36).

## Footnotes

^*^This is a point to take care with as many authors have opposite conventions for defining the adjacency matrix. Note also that the mean in-degree is always equal to the mean out-degree, so we can refer simply to the mean degree of a digraph.
