# Supplementary material for: Strong connectivity in real directed networks
Source: Proc Natl Acad Sci U S A. 2023 Mar 16;120(12):e2215752120. doi: 10.1073/pnas.2215752120 (PMC10041124; doi:10.1073/pnas.2215752120)
Supplement: Supplementary file 1 — Appendix 01 (PDF) [file pnas.2215752120.sapp.pdf]

Supplementary Material: Strong Connectivity in Real Directed Networks

Niall Rodgers [1,2]

School of Mathematics[1] and Topological Design Centre for Doctoral Training[2], University of Birmingham, Birmingham B15 2TT, United Kingdom

Peter Tiňo [3]

School of Computer Science[3], University of Birmingham, Birmingham B15 2TT, United Kingdom

Samuel Johnson [1,4]

School of Mathematics[1], University of Birmingham, Birmingham B15 2TT, United Kingdom and The Alan Turing Institute[4], British Library, 96 Euston Rd, London NW1 2DB, United Kingdom

A Level Distribution of Example Real Networks

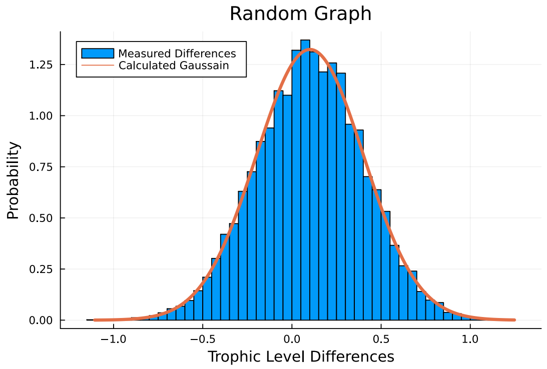

(a) ER random graphs with $N = 1000$ and $\langle k \rangle = 10$

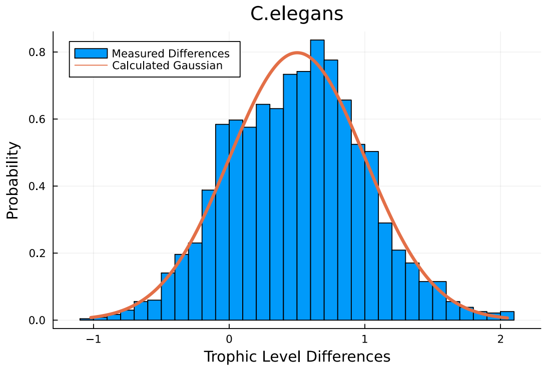

(b) *C.Elegans* connectome (S36)

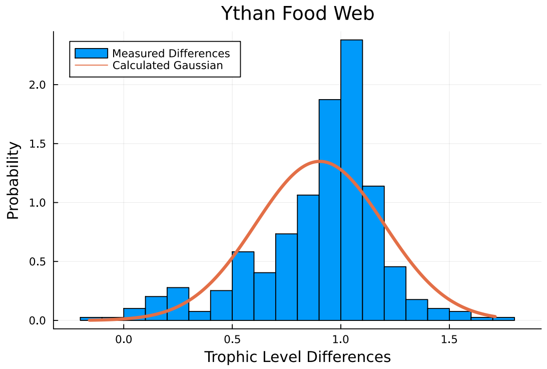

(c) Ythan Estuary Food Web

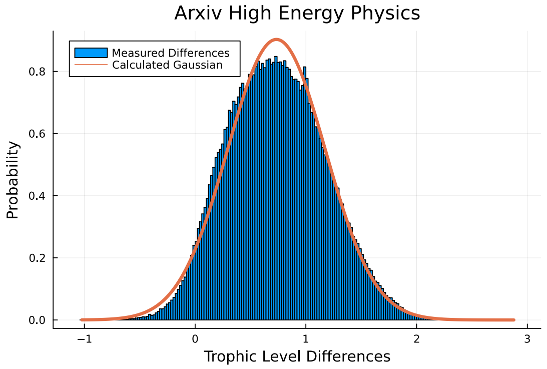

(d) Arxiv CItaion Network of High energy Physics with approx 35,000 nodes (S37). Downloaded from (S38).

Figure S1: Level difference distributions of real networks of a variety of sizes and function which can roughly be approximated as Gaussian's.

We show the level difference distributions for a variety of real world networks, figure S1. The Gaussian approximation is calculated from using mean, $\overline{z}$ and standard deviation $\overline{z}\eta$ as defined in the main text and as in (S3). All the distributions are reasonably well approximated by a Gaussian. The random graph, figure S1a is well approximated even though it has no hierarchical structure and so is the *C.elegans* connectome figure S1b which has a more complex structure. The approximation is not perfect for all networks for the small food web, figure S1b, as the network is very coherent the peak at one is larger than the Gaussian predicts and the network is quite small so we expect fluctuations and noise. However the approximation works well on very large networks such as the Arxiv Higher Energy Physics citation network.This network is very structured as more popular papers are cited more and it has an ordering imposed by time as you can only cite papers written before you. However the trophic level distribution is still well approximated by a Gaussian which depends only on F.

B Importance of Attacks on Backwards Edges for Dynamical Processes

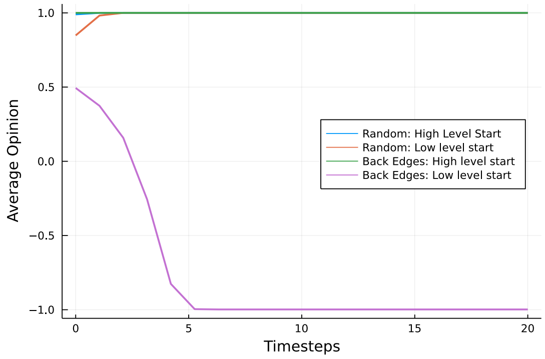

(a) Majority Vote Dynamics with Opinion Inertia on ER random graphs with $N = 1000$ and $\langle k \rangle = 10$ with 20% of most backwards edges targeted or random edges targeted with different starting locations in the hierarchy of with a fifth of nodes taking a new opinion.

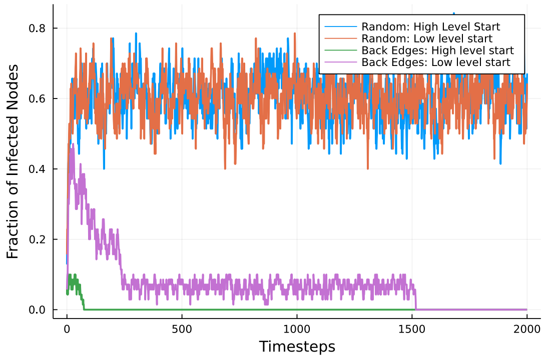

(b) Spread of infection with time in SIS model on High School Social Network (S39) with 20% of most backwards or random edges targeted with different starting locations in the hierarchy and an initial infection in 5% of the students. $pI = 0.2$, $pR = 0.1$.

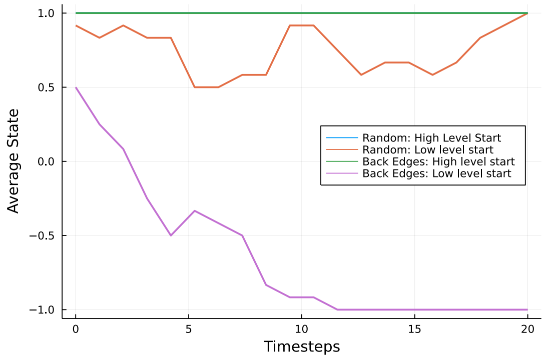

(c) Voter Model on Trade Network (S40) with 33% of most backwards or random edges targeted with different starting locations in the hierarchy and 5% of nodes taking the new state.

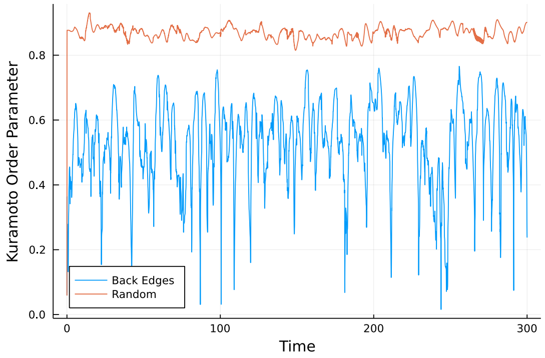

(d) Synchronisation of Continuous Kuramoto Oscillators on the *C.Elegans* connectome (S36) with 20% of most backwards edges targeted and random edges targeted starting from random phase between zero and 2π.

Figure S2: Dynamics of Real-World Networks after targeted attack on Backwards edges and Random Edge Attacks. All real-world data sets can be found at (S25) or (S40) for the trade network.

Strong connectivity is very important for many real-world networks and their dynamics. For illustrative purposes we demonstrate how a spreading process defined by a Susceptible-Infected-Susceptible (SIS) model dynamics; Opinion formation governed by Majority Vote Dynamics; changes in states of nodes governed by the the Voter Model and Synchronisation of Continuous Kuramoto Oscillators

all change after a targeted attack on the backwards edges calculated using Trophic Analysis and random attack. These are all demonstrated in figure S2.

Majority vote is a very simple model of opinion formation which is demonstrated in figure S2a. Each agent in the model is given an opinion and then updates their opinion if the majority of their neighbours have a different opinion. It is very simple but shares some similarities with other discrete dynamics widely used in complex networks such as the SIS epidemic model (S41), Hopfield neural network model (S21), the Ising model of magnetisation (S42) and the Moran process describing evolution of populations (S43). The simplest way to define majority vote dynamics is a system where there are two opinions denoted as +1 and -1. Each agent holds either of the two options and updates their state according to the majority opinion of their neighbours. In the case of a draw the new state can be randomly selected or remain in the previous state. We choose to fix the system in the previous state to prevent random opinions filtering through the system from the nodes with in-degree 0 when the backwards edges are removed. This update rule can be written as

$$S_i(t + \Delta t) = \text{sgn}\left(\sum_j A_{ij} S_j(t) + \delta S_i(t)\right). \tag{S1}$$

Where δ is a small positive constant less than one to account for balanced opinions. We also update the state of the system in parallel. In this setup, for maximum simplicity, we take an ErdősRényi random graph, with $N = 1000$ and $\langle k \rangle = 10$, and give one fifth of the nodes a new opinion, labelled -1, and observe how that opinion spreads over time. This could represent a political belief or taking part in a social trend. Figure S2a shows that how the opinion forms depends on where in the hierarchy the opinion starts and how much feedback there is in the system driven by the backwards edges. When the new opinion starts at the bottom of the hierarchy and the backwards edges have been attacked it can spread through the whole system and it becomes a majority opinion amongst the nodes. When the opinions starts at the bottom of the hierarchy after a random attack it can still slightly spread up through the system but since there is still feedback in the system the affect of the new opinion is damped so it quickly dies out. When the opinions starts at the top of the system and the back edges have been attacked it can not propagate back down the system so the new trend quickly dies out. When the opinions starts at the top of the system after a random attack it is also quickly replaced by the opinion from the lower trophic level nodes. This also demonstrates that hierarchical structure can be found and exploited even in networks like random graphs where little structure is expected.

The SIS model, figure S2b, is a simple spreading process which could be imagined to represent in the simplest way the spreading of an infection which you can catch multiple times and lack immunity like the common cold or sexually transmitted disease; the spreading of a meme in a social network; selling in a trading network or activation in a neural network. In this model each node can either be susceptible or infected. If a node is susceptible it becomes infected if any of its in-neighbours are infected with probability pI, at each time step. If a node is infected it loses the infection and transitions to the susceptible state with probability pR. There are many possible variations of this model however we use the simplest case for demonstration (S44). Using parallel updates we start the system with 5% of the nodes infected and the rest susceptible. This is shown in figure S2b where the dynamics take place on a High School Social Network. If random edges are removed the backwards edges remain and then the infection can cycle round the network and the infection becomes endemic. If the network is strongly connected the final state is not affected by where in the the network the infection appears however the initial spread can be affected by where

in the hierarchy it starts. When the backwards edges are removed there is no strongly connected component to maintain the infection so it eventually dies out. However the hierarchy induces an asymmetry in the network. If the infection starts in the low trophic level nodes it can spread through a large part of the network before it dies out so many nodes see the infection while if the infection begins in the high level nodes it has nowhere to spread to so quickly dies out, figure S2b. This is very important to understand as each of these scenarios can have very different consequences depending on the nature of the spreading agent and the system in question.

A voter model, figure S2c, is another simple model which can represent opinion formation or general updating of states of the agents in a network (S45). We take two discrete states labelled $+1$ and -1 and update the system in parallel such that at each time step a node selects one of it's in-neighbours at random and copies the state of the chosen neighbour. This model is very simple however variants of this model can be used in modelling real voting processes (S46), economics (S47) and chemistry (S48; S49). In our example dynamics we simulate the simple voter model on a trade network. Where the new state can represent any relevant binary change in the function of an entity for example if the agent suffers a delay or shortage in production or begins selling off particular assets. We start the system with 5% of the nodes in the new state and destroy one third of the edges, more edges have to be attacked due to the network being small and dense as well as quite incoherent. When the perturbation is made to the high level nodes the new state can not take hold and the system maintains it's previous state, figure S2c. However when the new state is introduced to the low level nodes it can gain a foothold. When it is presented at the low level nodes after a random attack it survives for some time before disappearing while after an attack on the backwards edges the new state is able to overtake the entire system.

The Kuramoto model is a very important model of synchronisation (S50) used in a wide variety of setting in particular in Neuroscience (S51; S52). In figure S2d we simulate Kuramoto oscillators on the neural structure of the nematode *C.Elegans* after random and targeted attack. It can be treated analytically in simple network topologies but in complex networks the model must be solved numerically. We use NetworkDyanmics.jl (S53) to solve the system of differential equations used in our variant of the model. Each oscillator has a phase, θ, which evolves according to the equation

$$\frac{d\theta_i}{dt} = \frac{K}{k_i^{\text{in}}} \sum_{j=1}^{N} A_{ji} \sin(\theta_j - \theta_i) + \omega_i. \tag{S2}$$

Where K is the coupling constant, k_i^{in} is the in-degree of node i and ω_i is the natural frequency of node i. We use the form normalised by in-degree so that the oscillators update at similar rates even if they have many input nodes. K is taken to be 50 to ensure synchronisation and the natural frequency of each node is drawn from a normal distribution with mean 0 and standard deviation 1. The synchronisation of the oscillators is shown in figure S2d and measured by the order parameter

$$r = \frac{1}{N} |\sum_{i=1}^{N} e^{i\theta_i}|. \tag{S3}$$

This reaches 1 when all the oscillators are fully in phase. Starting from each oscillator having an initial phase between 0 and 2π under a random attack the system is still able to synchronise. However when the backwards edges are attacked the synchronisation is less strong, S2d, and the system is disrupted.

These results tie into existing literature on directionality, spreading (S54) and epidemic thresholds in directed networks (S55). Where the epidemic threshold can be considered a function of

the spectral radius and driven by the directionality as measured by the fraction of bidirectional edges (S55). Our results are in agreement with this as a bidirectional edge pair must count at least one edge where the trophic level difference is less than or equal to zero. We extend the definition of directionality to make it more general and complete rather than simply the fraction of edges which are bidirectional (S55). Our results can also be restated in terms of the spectral radius of the adjacency matrix as there is an analytical estimate of the spectral radius as a function of F (S3). Similar results demonstrating the affect of directionality and hierarchy on the performance of Hopfield-like neural networks can be found in (S21). The results demonstrate the importance of the backwards edges to the system across a variety of dynamics and scales which we expect to hold in a variety of other systems. However it should be stressed that the importance of the backwards edges to dynamics depends on a variety of factors. Firstly the impact of attacking the strongly connected component depends on the initial size and distribution of that component as if the component is initially very small destroying it may have little affect. Degree distribution can also interplay very strongly with dynamics both in the sense that hub nodes of very high degree can play in important role in controlling the dynamics and that directed networks can potentially have many nodes which have in-degree zero (S34). Nodes which have in-degree zero have no input from the system so internally set their own state so can play a large role in controlling the system dependant on their placement in the hierarchy, out-degree and the specifics of their internal dynamics. In addition this could be repeated with another measure of the hierarchical ordering and targeting of the backwards edges however you could not analytically estimate the number of edges you would need to target before enumerating all the backwards edges and would also lack the link to the global directionality.

C Network Data-sets

The majority of the networks used in this study are stored at (S25) where they were previously used in the results surrounding the relationship between trophic structure and spectral radius (S17). Information about the original sources of the networks as well as additional network information about the structure can be found in the supplementary information of (S17) or online at (S25).

We also supplement this data-set with some additional networks from (S22), `https://networks.skewed.de/`, in order to sample the parameter space as best as we could which are listed in the table below. The links provide more detailed information about the network structure and origin and the original source as listed in (S22) is referenced in the last column.

Network Name	Retrieved From	Source
FAA Preferred Routes	https://networks.skewed.de/net/faa_routes	(S56)
Dutch school friendships (all 6 connected networks)	https://networks.skewed.de/net/dutch_school	(S57)
Abu Sayyaf kidnappings	https://networks.skewed.de/net/kidnappings	(S58)
Papuan gift-giving	https://networks.skewed.de/net/moreno_taro	(S59)
Swingers and parties	https://networks.skewed.de/net/swingers	(S60)
Email network (Uni. R-V, Spain, 2003)	https://networks.skewed.de/net/uni_email	(S61)
Political blogs network	https://networks.skewed.de/net/polblogs	(S62)

D Strong Connectivity By Network Type

In the main text we give the result for predicting the strong connectivity for real networks where all the networks of different types are shown in the same figure. Here we break down the networks by the categories included in the data set (S25; S17). All the networks are collected in different ways so there may be uncertainty associated with how well networks of each type represent the underlying real world system.

Food-webs, figure S3, are unlikely to be strongly connected as expected as they have a very hierarchical structure as result of the interactions between species and the flow of energy up the food chain. It is interesting to note that some food webs have high degrees where previous work on random directed networks (S4) would expect the network to be strongly connected.

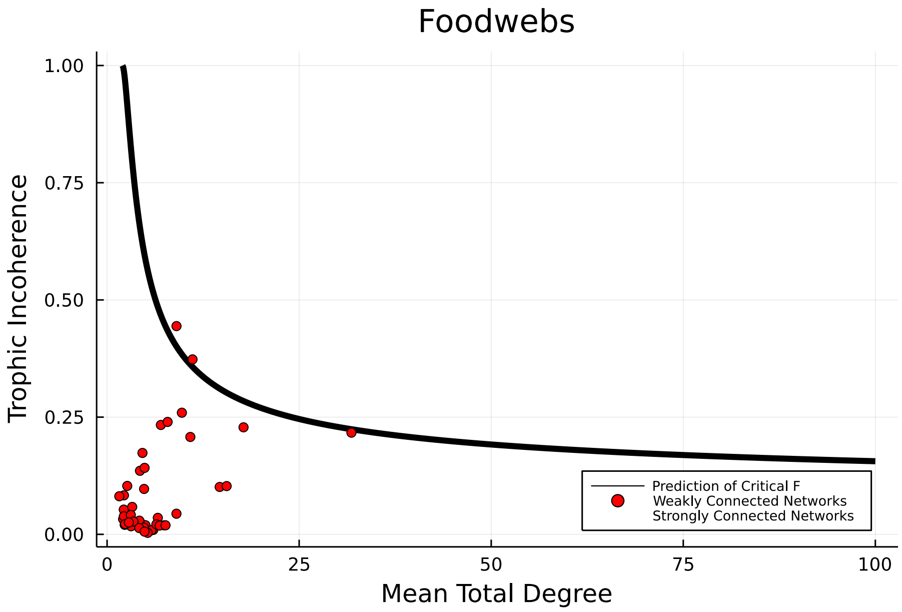

Figure S3: Prediction of Strong Connectivity in Food webs from (S25)

The genetic networks in our data set, figure S4, are all very coherent and lack a strongly connected component.

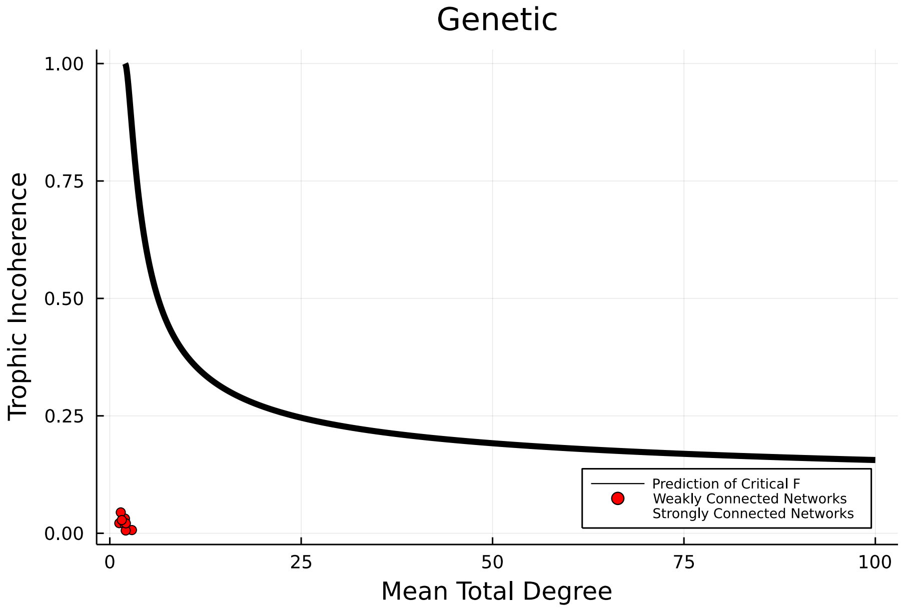

Figure S4: Prediction of Strong Connectivity in Genetic Networks from (S25)

The singular language network, figure S5, is quite incoherent but lies close to the transition line.

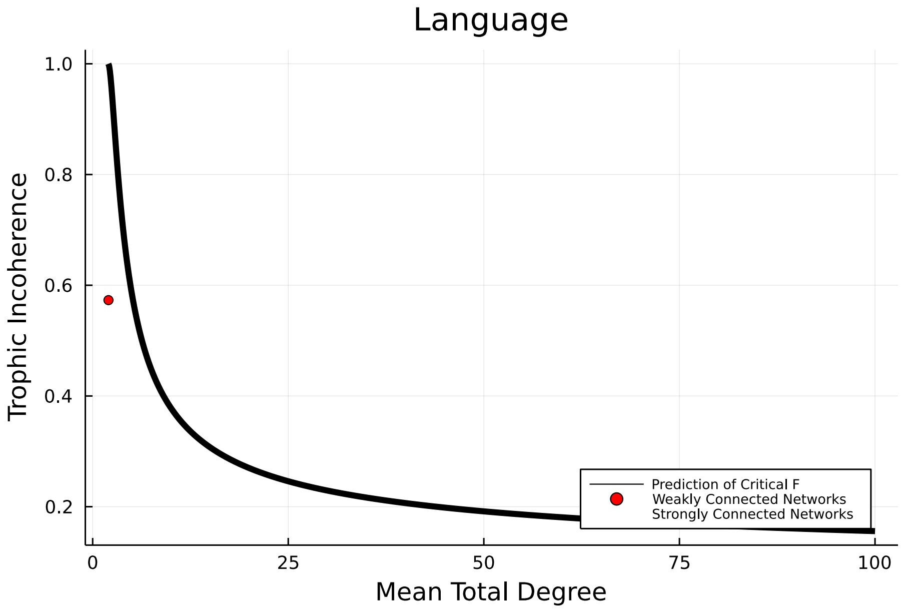

Figure S5: Prediction of Strong Connectivity in Language Networks from (S25)

The metabolic networks, S6, all lie very close to the transition line and are very incoherent so have a little global hierarchical structure but still enough to make our analysis relevant.

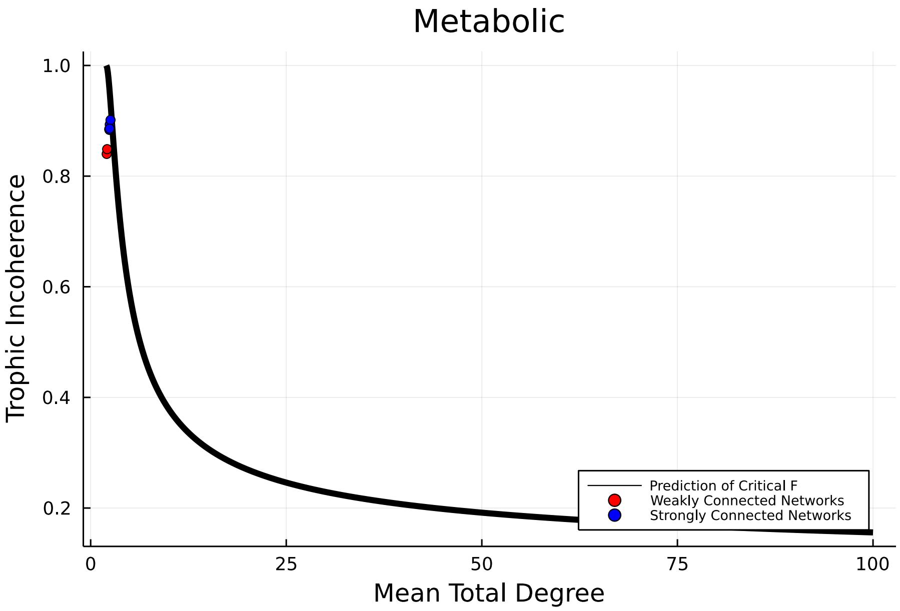

Figure S6: Prediction of Strong Connectivity in Metabolic Networks from (S25)

There is large variation in the neural network data set, figure S7, this is due to the variety of data types and methods employed in this field. This data includes the connectome of *C.Elegans* as well as functional brain networks for a variety of species.

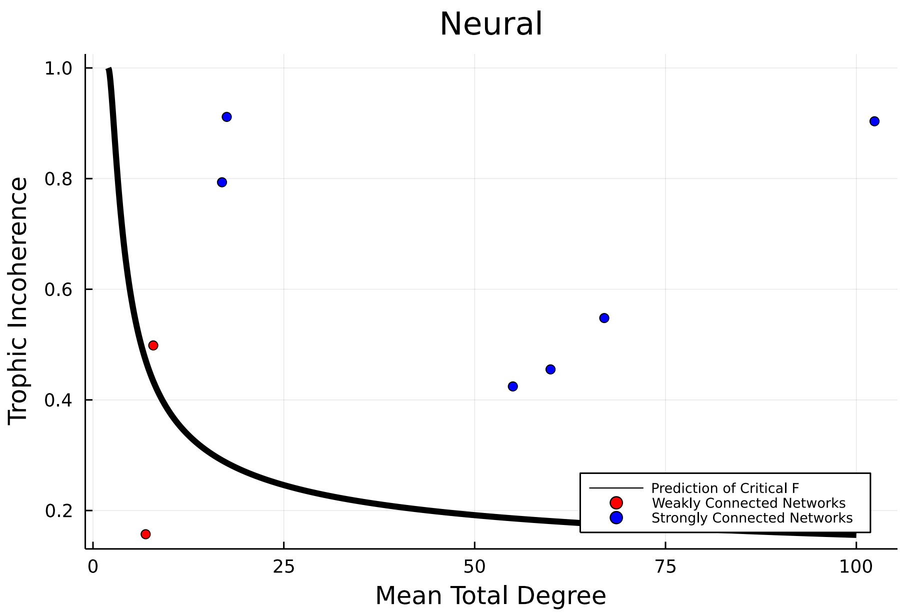

Figure S7: Prediction of Strong Connectivity in Neural Networks from (S25)

The social network comprised of in-person friendships all lie close to the transition line, figure S8, between strong and weak connectivity. Again social networks are a type of real network where you might initially expect there to be very little hierarchy and ordering.

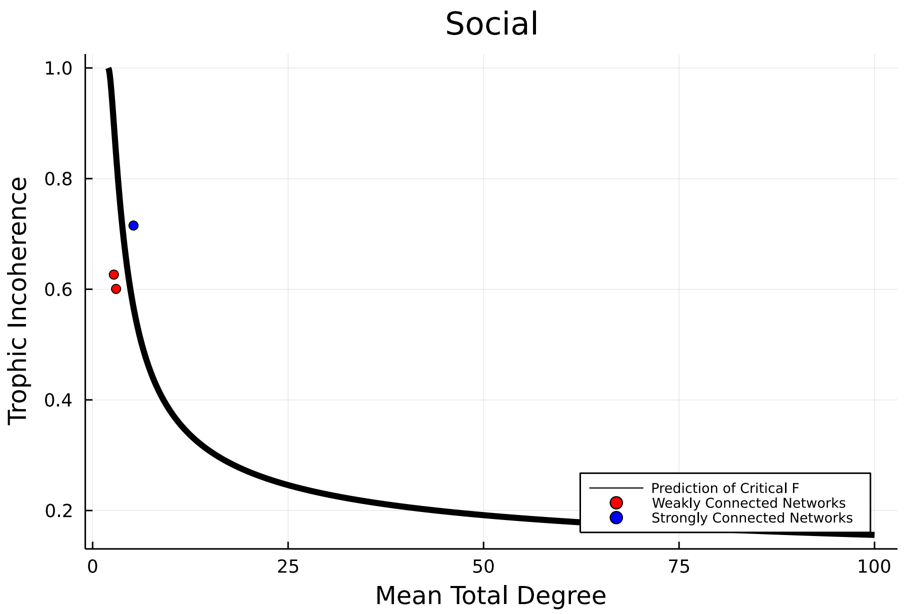

Figure S8: Prediction of Strong Connectivity in Social Networks from (S25)

The trade networks in our data set, figure S9, are all in a similar region which is mostly strongly connected but still exhibits some hierarchy.

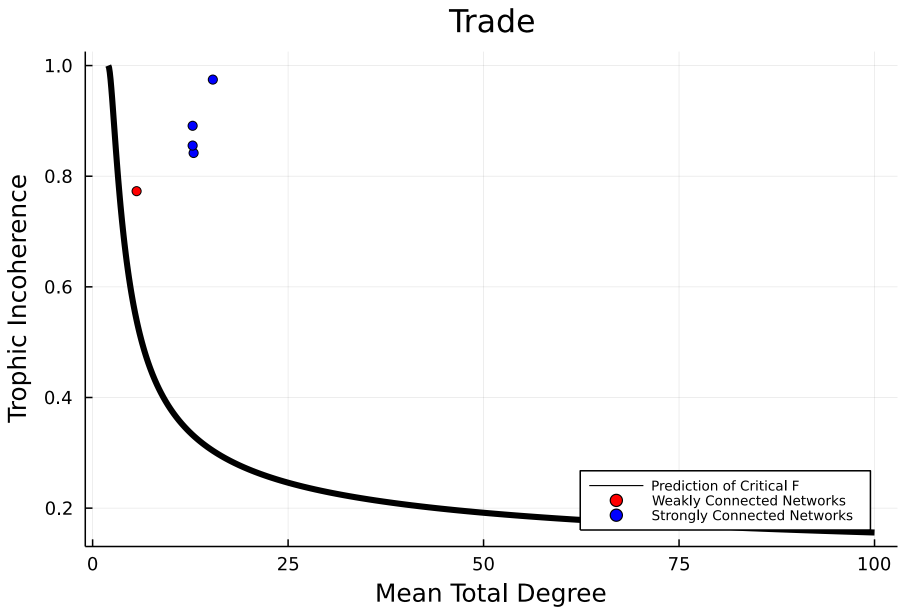

Figure S9: Prediction of Strong Connectivity in Trade Networks from (S25)

E Branching Factor for all Networks

The branching factor is shown for all the from (S25) networks in figure S10. Again it is clear the the transition is missed when not considering the hierarchy and methods derived from random graphs (S4) are not enough to explain the behaviour of real networks.

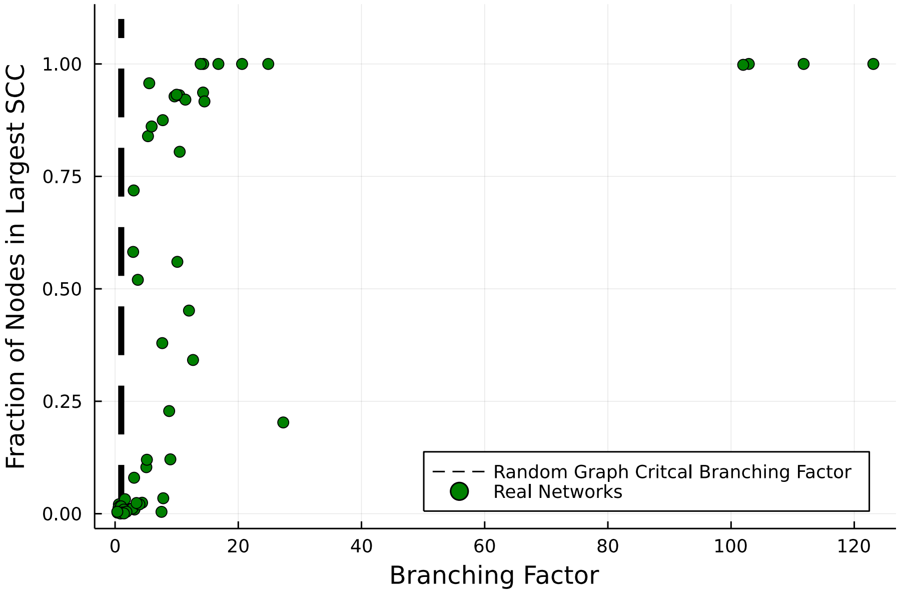

Figure S10: Prediction of Strong Connectivity using the Branching Factor for Real Networks (S25).

References

[S1] Li M, et al. (2021) Percolation on complex networks: Theory and application. *Physics Reports* 907.

[S2] Johnson S (2020) Digraphs are different: why directionality matters in complex systems. *Journal of Physics: Complexity* 1(1):015003.

[S3] MacKay RS, Johnson S, Sansom B (2020) How directed is a directed network? *Royal Society Open Science* 7(9):201138.

[S4] Boguñá M, Serrano M (2005) Generalized percolation in random directed networks. *Physical Review E - Statistical, Nonlinear, and Soft Matter Physics* 72(1).

[S5] Asllani M, Lambiotte R, Carletti T (2018) Structure and dynamical behavior of non-normal networks. *Science Advances* 4(12).

[S6] Duan C, Nishikawa T, Eroglu D, Motter AE (2022) Network structural origin of instabilities in large complex systems. *Sci. Adv* 8:8310.

[S7] Coupette F, Schilling T (2022) Exactly solvable percolation problems. *Physical Review E* 105(4):044108.

[S8] Johnson S, Domínguez-García V, Donetti L, Muñoz MA (2014) Trophic coherence determines food-web stability. *Proceedings of the National Academy of Sciences of the United States of America* 111(50):17923–17928.

[S9] May RM (1972) Will a large complex system be stable? *Nature* 238(5364).

[S10] Levine S (1980) Several measures of trophic structure applicable to complex food webs. *Journal of Theoretical Biology* 83(2).

[S11] Klaise J, Johnson S (2017) The origin of motif families in food webs. *Scientific Reports* 7(1).

[S12] Klaise J, Johnson S (2016) From neurons to epidemics: How trophic coherence affects spreading processes. *Chaos* 26(6).

[S13] Pagani A, et al. (2019) Resilience or robustness: Identifying topological vulnerabilities in rail networks. *Royal Society Open Science* 6(2).

[S14] Pagani A, Meng F, Fu G, Musolesi M, Guo W (2020) Quantifying Resilience via Multiscale Feedback Loops in Water Distribution Networks. *Journal of Water Resources Planning and Management* 146(6).

[S15] Pilgrim C, Guo W, Johnson S (2020) Organisational Social Influence on Directed Hierarchical Graphs, from Tyranny to Anarchy. *Scientific Reports* 10(1).

[S16] Erdös P, Rényi A (1959) On random graphs I. *Publicationes Mathematicae* 6.

[S17] Johnson S, Jones NS (2017) Looplessness in networks is linked to trophic coherence. *Proceedings of the National Academy of Sciences of the United States of America* 114(22).

[S18] De Bacco C, Larremore DB, Moore C (2018) A physical model for efficient ranking in networks. *Science Advances* 4(7).

[S19] Kichikawa Y, Iyetomi H, Iino T, Inoue H (2019) Community structure based on circular flow in a large-scale transaction network. *Applied Network Science* 4(1).

[S20] Iyetomi H, Ikeda Y, Mizuno T, Ohnishi T, Watanabe T (2017) International Trade Relationship from a Multilateral Point of View in *The 6th International Conference on Complex Networks & Their Applications.* (Lyon, France), pp. 253–255.

[S21] Rodgers N, Tio P, Johnson S (2022) Network hierarchy and pattern recovery in directed sparse Hopfield networks. *Physical Review E* 105(6):064304.

[S22] Peixoto TP ((accessed December, 2022)) *The Netzschleuder network catalogue and repository.* `https://networks.skewed.de/`.

[S23] Sun J, Ajwani D, Nicholson PK, Sala A, Parthasarathy S (2017) Breaking cycles in noisy hierarchies in *WebSci 2017 - Proceedings of the 2017 ACM Web Science Conference.*

[S24] Brin S, Page L (1998) The anatomy of a large-scale hypertextual web search engine. *Computer Networks and ISDN Systems* 30(1):107 – 117. Proceedings of the Seventh International World Wide Web Conference.

[S25] Johnson S ((accessed October, 2020)) *www.samuel-johnson.org Data Repository.* `https://www.samuel-johnson.org/data`.

[S26] Rodgers N, Tio P, Johnson S (2022) Influence and Influenceability: Global Directionality in Directed Complex Networks. *ArXiv* arXiv:2210.12081.

[S27] Verbavatz V, Barthelemy M (2021) From one-way streets to percolation on random mixed graphs. *Physical Review E* 103(4).

[S28] Schick RS, Lindley ST (2007) Directed connectivity among fish populations in a riverine network. *Journal of Applied Ecology* 44(6).

[S29] Hock K, Wolff NH, Condie SA, Anthony KR, Mumby PJ (2014) Connectivity networks reveal the risks of crown-of-thorns starfish outbreaks on the Great Barrier Reef. *Journal of Applied Ecology* 51(5).

[S30] Hock K, et al. (2016) Controlling range expansion in habitat networks by adaptively targeting source populations. *Conservation biology : the journal of the Society for Conservation Biology* 30(4).

[S31] Hock K, et al. (2017) Connectivity and systemic resilience of the Great Barrier Reef. *PLoS Biology* 15(11).

[S32] Breskin I, Soriano J, Moses E, Tlusty T (2006) Percolation in living neural networks. *Physical Review Letters* 97(18).

[S33] Soriano J, Martínez MR, Tlusty T, Moses E (2008) Development of input connections in neural cultures. *Proceedings of the National Academy of Sciences of the United States of America* 105(37).

[S34] Wright EA, Yoon S, Ferreira AL, Mendes JF, Goltsev AV (2019) The central role of peripheral nodes in directed network dynamics. *Scientific Reports* 9(1).

[S35] Fairbanks J, et al. (2021) Juliagraphs/graphs.jl: an optimized graphs package for the julia programming language.

[S36] White JG, Southgate E, Thomson JN, Brenner S (1986) The structure of the nervous system of the nematode Caenorhabditis elegans . *Philosophical Transactions of the Royal Society of London. B, Biological Sciences* 314(1165).

[S37] Leskovec J, Kleinberg J, Faloutsos C (2005) Graphs over time: Densification laws, shrinking diameters and possible explanations in *Proceedings of the ACM SIGKDD International Conference on Knowledge Discovery and Data Mining.*

[S38] Leskovec J, Krevl A (2014) SNAP Datasets: Stanford large network dataset collection (`http://snap.stanford.edu/data`).

[S39] Rossi RA, Ahmed NK (2015) The network data repository with interactive graph analytics and visualization in *Proceedings of the National Conference on Artificial Intelligence*. Vol. 6.

[S40] De Nooy W, Mrvar A, Batagelj V (2018) *Exploratory Social Network Analysis with Pajek.*

[S41] Volkening A, Linder DF, Porter MA, Rempala GA (2020) Forecasting elections using compartmental models of infection. *SIAM Review* 62(4).

[S42] Kim M, Yook SH (2021) Majority-vote model with degree-weighted influence on complex networks. *Physical Review E* 103(2).

[S43] Moinet A, Barrat A, Pastor-Satorras R (2018) Generalized voterlike model on activity-driven networks with attractiveness. *Physical Review E* 98(2).

[S44] Hethcote HW (1989) Three Basic Epidemiological Models.

[S45] Redner S (2019) Reality-inspired voter models: A mini-review.

[S46] Fernández-Gracia J, Suchecki K, Ramasco JJ, San Miguel M, Eguíluz VM (2014) Is the Voter Model a Model for Voters? *Physical Review Letters* 112(15).

[S47] Kirman A (1993) Ants, rationality, and recruitment. *Quarterly Journal of Economics* 108(1).

[S48] Fichthorn K, Gulari E, Ziff R (1989) Noise-induced bistability in a Monte Carlo surface-reaction model. *Physical Review Letters* 63(14).

[S49] Considine D, Redner S, Takayasu H (1989) Comment on noise-induced bistability in a Monte Carlo surface-reaction model.

[S50] Acebrón JA, Bonilla LL, Vicente CJ, Ritort F, Spigler R (2005) The Kuramoto model: A simple paradigm for synchronization phenomena. *Reviews of Modern Physics* 77(1).

[S51] Bick C, Goodfellow M, Laing CR, Martens EA (2020) Understanding the dynamics of biological and neural oscillator networks through exact mean-field reductions: a review. *Journal of Mathematical Neuroscience* 10(1).

[S52] Cumin D, Unsworth CP (2007) Generalising the Kuramoto model for the study of neuronal synchronisation in the brain. *Physica D: Nonlinear Phenomena* 226(2).

[S53] Lindner M, et al. (2021) NetworkDynamics.jlComposing and simulating complex networks in Julia. *Chaos: An Interdisciplinary Journal of Nonlinear Science* 31(6).

[S54] Zhu YX, et al. (2014) Influence of reciprocal links in social networks. *PLoS ONE* 9(7).

[S55] Li C, Wang H, Van Mieghem P (2013) Epidemic threshold in directed networks. *Physical Review E - Statistical, Nonlinear, and Soft Matter Physics* 88(6).

[S56] FAA ((accessed 2010)) *Air traffic control system command center.* `http://www.fly.faa.gov/`.

[S57] Snijders TA, van de Bunt GG, Steglich CE (2010) Introduction to stochastic actor-based models for network dynamics. *Social Networks* 32(1).

[S58] Gerdes LM, Ringler K, Autin B (2014) Assessing the Abu Sayyaf Group's Strategic and Learning Capacities. *Studies in Conflict and Terrorism* 37(3).

[S59] Schwimmer EG (1970) Ph.D. thesis (University of British Columbia).

[S60] Niekamp AM, Mercken LA, Hoebe CJ, Dukers-Muijrers NH (2013) A sexual affiliation network of swingers, heterosexuals practicing risk behaviours that potentiate the spread of sexually transmitted infections: A two-mode approach. *Social Networks* 35(2).

[S61] Guimerà R, Danon L, Díaz-Guilera A, Giralt F, Arenas A (2003) Self-similar community structure in a network of human interactions. *Physical Review E - Statistical Physics, Plasmas, Fluids, and Related Interdisciplinary Topics* 68(6).

[S62] Adamic LA, Glance N (2005) The political blogosphere and the 2004 U.S. Election: Divided they blog in *3rd International Workshop on Link Discovery, LinkKDD 2005 - in conjunction with 10th ACM SIGKDD International Conference on Knowledge Discovery and Data Mining.*
